# A feasible and effective method for restoring patency of a biliary T-tube sinus tract

**DOI:** 10.1308/003588412X13373405385214f

**Published:** 2012-07

**Authors:** M Wang, Z Fan, S Huang

**Affiliations:** Second Affiliated Hospital of Nanjing Medical University,China

## BACKGROUND

T-tube placement following bile duct exploration remains commonplace.^1^ Via an unobstructed T-tube tract, choledochoscopic removal of retained biliary stones has become a well established mode of treatment, having been used as early as 1982.^2^ However, T-tubes or reinserted straight drainage tubes after choledochoscopy are displaced frequently by accident, which results in sinus tract occlusion.^3^ Generally, an emergency reoperation or endoscopic retrograde cholangiopancreatography (ERCP) may be necessary if the T-tube tract cannot be recanalised promptly. We describe a new technique to reinsert the drainage tube and recanalise the T-tube sinus tract before its complete closure.

## TECHNIQUE

**Figure 1:**
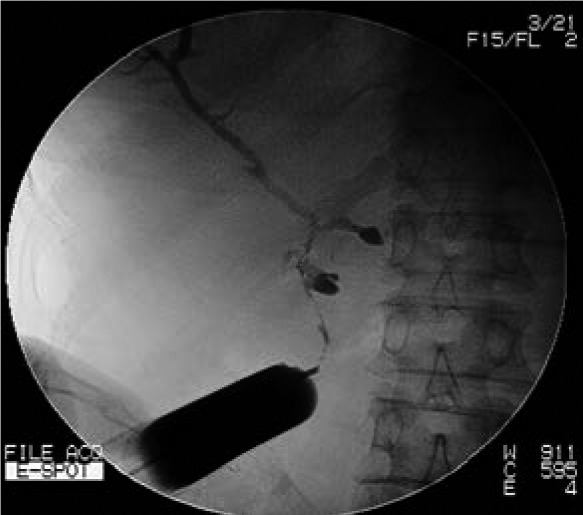
Iohexol contrast media is injected with pressure through the narrow opening of the sinus on the skin to identify the location of the T-tube tract.

**Figure 2:**
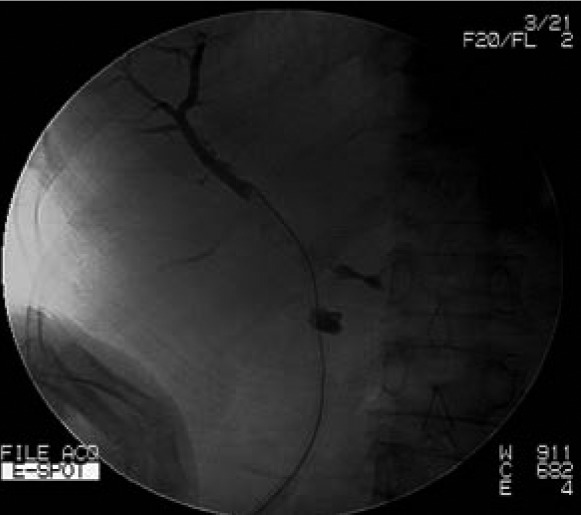
A soft guidewire is inserted into the sinus tract until it reaches the common bile duct.

**Figure 3:**
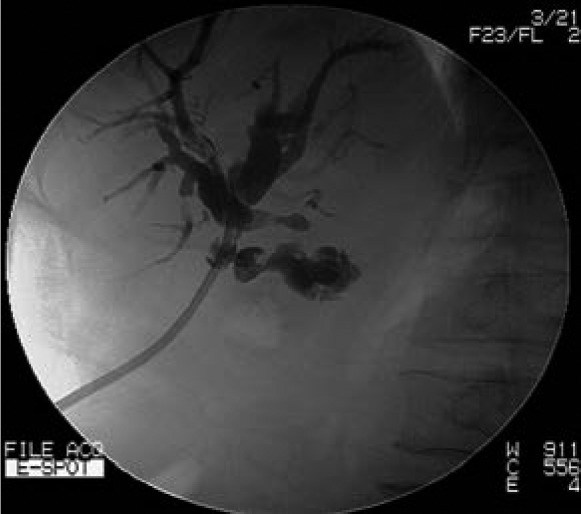
The T-tube sinus tract, intrahepatic ducts and common bile duct following fistula dilation and insertion of a stomach tube along the guidewire.

Iohexol contrast media is injected with pressure through the cutaneous opening of the T-tube sinus tract. Post-contrast imaging helps to identify the location of T-tube tract (Fig 1). Using x-ray fluoroscopy, a soft guidewire is inserted into the sinus track until it reaches the common bile duct (Fig 2). A biliary balloon dilator is introduced along the guidewire and dilation is then performed. A 16F stomach tube is passed along the fistula into the common bile duct with the guidewire (Fig 3). Residual stones in the bile duct can be removed in the following 1–2 weeks using choledochoscopy.

## DISCUSSION

The technique of drainage tube reinsertion using x-ray fluoroscopy is a safe and effective method for restoring the patency of a T-tube sinus tract and may avoid reoperation or ERCP.
